# *P16* DNA Methylation Coupled with Somatic Copy Number Variations in the Development of Gastric Carcinomas

**DOI:** 10.3390/cancers18101605

**Published:** 2026-05-15

**Authors:** Ziqian Yang, Jing Zhou, Lewen Deng, Juanli Qiao, Liankun Gu, Dajun Deng

**Affiliations:** Key Laboratory of Carcinogenesis and Translational Research (MOE/Beijing), Division of Etiology, Peking University Cancer Hospital and Institute, Beijing 100142, China; 2311210630@stu.pku.edu.cn (Z.Y.); lewen.deng@pku.org.cn (L.D.); qiaojl@bjmu.edu.cn (J.Q.);

**Keywords:** CDKN2A, *P16*, somatic copy number variation (SCNV), DNA methylation, gastric cancer, metastasis

## Abstract

**Background/Objectives:** Tumor suppressor genes are often inactivated by genetic and epigenetic mechanisms. However, whether genetic alterations of these genes, including *CDKN2A*/*P16*, are coupled with epigenetic changes in cancer development and progression is unknown. **Methods:** Freshly frozen gastric carcinoma (GC) samples, paired noncancer surgical margin (SM) samples, white blood cell (WBC) samples, and clinicopathological information were collected from 200 patients. The copy number (CN) of the *CDKN2A*/*P16* gene in these samples was determined by a P16-Light assay and normalized to that in white blood cells (WBCs). The DNA methylation level of the *P16* promoter in GC and SM samples was determined by a 115 bp *P16*-specific MethyLight assay. **Results:** Both the *P16* copy number and the DNA methylation level were significantly lower in GC samples than in SM samples (median, 1.94 vs. 2.14, *p* < 0.001 for *P16* CN; 0.0004 vs. 0.0013, *p* = 0.002 for *P16* methylation) and were associated with GC metastasis. The normalized *P16* copy number was significantly lower in GCs without vs. with *P16* methylation (*p* = 0.007). Similarly, more *P16* somatic copy number deletions (SCNdel) were detected in GCs without vs. with *P16* methylation (38.6% vs. 24.1%, *p* = 0.027). **Conclusions:** Somatic *P16* copy number variations are closely coupled with *P16* promoter DNA methylation during GC development. SCNdel and promoter DNA methylation complementarily inactivate *P16* in GC development and promote GC metastasis.

## Simple Summary

As one of the genes mostly changed in cancer genomes, *CDKN2A*/*P16* is often inactivated by copy number loss and promoter DNA methylation. It is unknown whether these two inactivation mechanisms are relevant. In this study, we determined the levels of *CDKN2A*/*P16* copy number and DNA methylation in gastric cancer (GC) and paired normal tissue samples from 200 patients. We found for the first time more *P16* deletions in GC tissues without vs. with *P16* methylation. Both *P16* copy number and DNA methylation levels are significantly decreased during GC development and are associated with GC metastasis. Our findings indicate that both somatic copy number deletion and promoter DNA methylation complementarily inactivate *CDKN2A*/*P16* in GC development and promote GC metastasis.

## 1. Introduction

*CDKN2A* encodes both P16 and P14 proteins. DNA methylation of CpG islands around the transcription start site (TSS-CGI) of the *P16* gene is prevalent in precancerous lesions across organs and drives cancer development and metastasis [1,2,3,4,5]. Our recent findings indicate that both *P16* somatic copy number deletion and amplification (SCNdel and SCNamp, respectively) are prevalent in precancerous esophageal squamous cell dysplasia and noncancerous surgical margin (SM) tissues from gastric carcinoma (GC) patients. *P16* SCNdel is significantly associated with poor prognosis in patients with ESCdys and GC, whereas *P16* SCNamp is associated with good prognosis in patients with these diseases [6,7,8]. Both genetic and epigenetic inactivation of the *P16* gene are frequent early driving events in cancer development in humans [9,10].

TSS-CGI hypermethylation not only epigenetically inactivates gene transcription [11] but also causes chromatin condensation. Because *P16* inactivation leads to dysfunction of the G1-S checkpoint in the cell cycle via the RB1 pathway [12,13], RB1 loss of function by *P16* inactivation may consequently cause replication stress and genome instability [14,15,16]. However, whether somatic copy number variations (SCNVs) are coupled with DNA methylation of the TSS-CGI of genes, including *P16*, has not been studied previously.

In this study, to investigate the relationship between *P16* SCNVs and DNA methylation in cancer development, we compared the *P16* SCNV frequency in GC and SM samples with and without *P16* TSS-CGI methylation from 200 patients. We revealed for the first time that *P16* SCNdel is more prevalent in gastric tissues, mainly in GCs, without *P16* methylation than in those with *P16* methylation, whereas *P16* SCNamp is more prevalent in gastric tissues, mainly in SMs, with *P16* methylation than in those without *P16* methylation. *P16* methylation and the SCNV complementarily promote GC development and metastasis.

## 2. Methods and Materials

### 2.1. Patients

The cohort was composed of 200 GC patients, including 140 males and 60 females; 106 and 94 patients with and without lymph metastasis, respectively, who underwent gastrectomy at the Peking University Cancer Hospital from January 2013 to November 2016. GC, SM (5 cm away from the main cancer mass, from the greater curvature on the distal side), and white blood cell (WBC) samples were freshly collected from these patients and stored in a freezer at −80 °C for approximately 8–10 years at the biobank of the hospital. No cancer cells were observed in these SM samples under an optical microscope. Clinicopathological information and overall survival data were collected. Among these 200 patients, 80 were included in our previously published study, in which only data on *P16* SCNV, but not *P16* methylation data, were available [8]. Detailed information for all 200 patients is listed in Data File S1 and summarized in Table 1. The Institute Review Board of the Peking University Cancer Hospital and Institute approved the study and the patients provided written informed consent to participate.

### 2.2. Preparation of Genomic DNA

Genomic DNA was extracted from the abovementioned frozen GC, SM, and WBC samples via the phenol/chloroform technique and used for *P16* copy number analysis. The GC and SM DNA samples were further modified with sodium bisulfite with an EZ DNA Methylation-Gold Kit (Zymo Research, Tustin, CA, USA) following the manufacturer’s instructions and used for *P16* methylation analysis.

### 2.3. Quantification of P16 Methylation Using the MethyLight Assay

An established 115 bp MethyLight assay [17] was used to quantify the proportion of methylated *P16* alleles in triplicate. The *COL2A1* gene, which contains no TSS-CGI, was used as an internal reference. When the copy number of methylated *P16* relative to *COL2A1* was greater than 2.74 × 10^−4^ in one of three PCR tubes for a bisulfite-modified DNA sample, the sample was defined as *P16* methylation-positive (P16M), *P16* methylation-negative otherwise (P16U).

### 2.4. Quantification of P16 Copy Number Using the P16-Light Assay

*CDKN2A*/*P16* copy number (CN) was quantified using droplet digital PCR based on P16-Light [8,18], in which the copy number of *GAPDH* was used as an internal reference gene.

### 2.5. Definitions of CDKN2A/P16 SCNamp and SCNdel

As we defined previously, the average CN of *P16* in WBCs from each patient was used as the diploid reference. The difference in the average *P16* copy number between the tested (GC or SM) sample and the paired WBC sample was calculated for each patient. When the difference in the copy number was statistically significant (*p* < 0.05) according to Student’s *t* test and the absolute fold change was greater than 20%, we defined the sample as *P16* SCNamp- or SCNdel-positive, as we previously reported [8].

### 2.6. Statistical Analysis

We used the Wilcoxon test or Mann–Whitney test to compare the proportion of methylated *P16* alleles and Student’s *t* test or chi-square test to compare *P16* copy number between different SM and GC samples or subgroups. Log-rank univariate analysis was used to compare patient overall survival between groups in the K–M analysis. All tests were two-sided, and a *p* value less than 0.05 indicated statistical significance.

## 3. Results

### 3.1. Basic Results of P16 SCNV and P16 Methylation Analyses in GC and Paired SM Samples from 200 Patients

**Figure 1:**
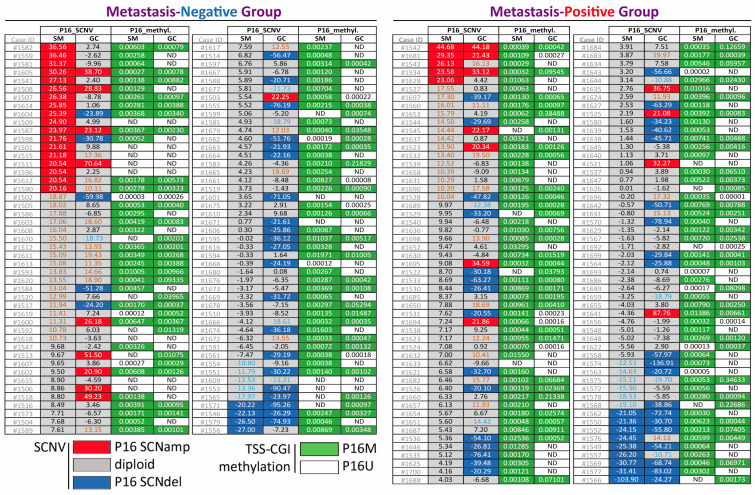
Detailed results of *P16* SCNV and methylation analyses for GC and SM samples from all 200 patients with and without metastasis. The exact differences in *P16* CNs between WBCs and GCs or SMs and *P16* methylation levels are listed for each GC or SM sample. SCNamp (marked in red) or SCNdel (marked in blue), somatic copy number amplification or deletion, respectively; P16M (marked in green) or P16U (marked in white), *P16* TSS-CGI methylation-positive or -negative, respectively.

The results of *P16* CN and TSS-CGI methylation analyses were obtained with P16-specific droplet digital PCR and MethyLight assays for GC, SM, and WBC samples from all 200 patients (Figure 1). A considerable frequency of *P16* SCNamp was observed in both SMs and GCs (11.5% vs. 10.5%). Moreover, *P16* SCNdel mostly occurred in GCs rather than SMs (30.5% vs. 6.5%, *p* < 0.001) (Table 1).

**Figure 2:**
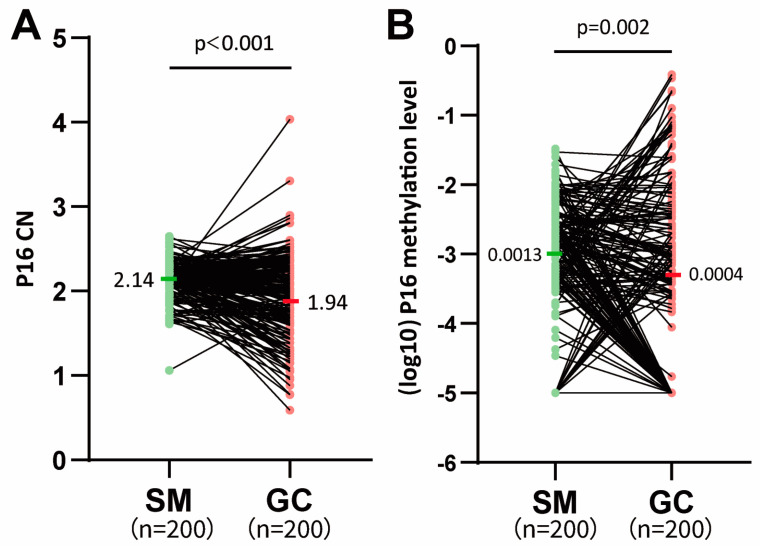
Comparison of *P16* CN and TSS-CGI methylation levels in GC and paired SM samples from 200 patients. (**A**) *P16* CN level (relative to WBC); (**B**) level of methylated *P16* (relative to *COL2A1*); GC and paired SM samples from the same patient are linked with a black line. The average *P16* CN and *P16* methylation values (median) are labeled.

The average *P16* CN value was significantly greater in SMs than in GCs (Figure 2A). Similarly, the percentage of *P16*-methylated samples and the overall prevalence of *P16* methylation were significantly greater in SMs than in GCs (81.0% vs. 56.0%, *p* < 0.001; median, 0.0013 vs. 0.0004, *p* = 0.002; Figure 2B), although the *P16* methylation level for P16M samples was slightly lower in SMs than in GCs (median, 0.0018 vs. 0.0020; Table 2).

### 3.2. P16 SCNamp Coupled with P16M, Whereas P16 SCNdel Coupled with P16U in Gastric Tissues

**Figure 3:**
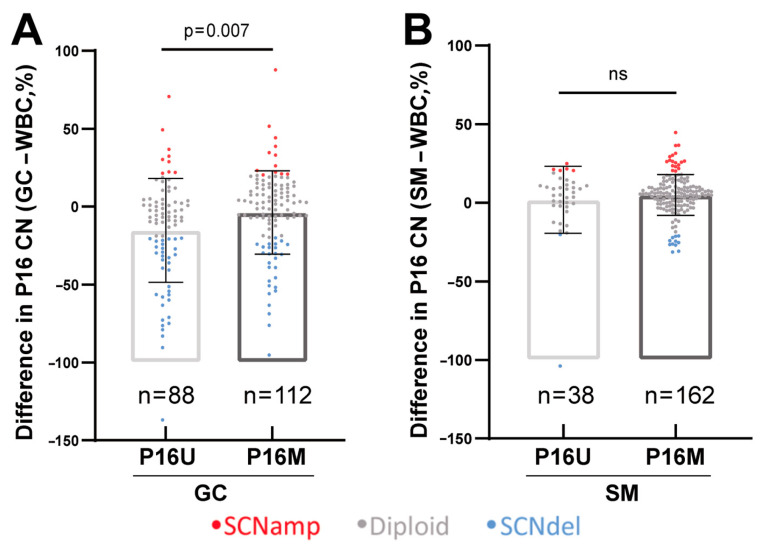
Comparison of differences in *P16* CN between gastric tissue samples with and without *P16* methylation from 200 patients. Percentage change in the *P16* CN in (**A**) GCs and (**B**) SMs relative to that in WBCs; the SCNV types are labeled with different colors. P16M and P16U, *P16* TSS-CGI methylation-positive and -negative, respectively. ns, not significant.

We further compared the level of *P16* CN (relative to that in WBCs) in GC and SM samples with and without *P16* methylation. We found that the average *P16* CN was significantly greater in P16M GC samples (*n* = 112) than in P16U GC samples (*n* = 88) (Figure 3A). A similar but nonsignificant difference was also observed in the SM samples (Figure 3B). Notably, more *P16* SCNdel was detected in P16U GC samples (34/88 = 38.6%) than in P16M GC samples (27/112 = 24.1%, *p* = 0.027). These results suggest that *P16* SCNdel is closely coupled with P16U, whereas *P16* SCNamp is coupled with P16M in gastric cancer tissues from GC patients.

### 3.3. P16 SCNVs and P16M in GC or SM Samples Are Complementarily Associated with GC Metastasis

Both the average *P16* CN value and the *P16* SCNamp-positive rate were significantly greater in SMs from patients without vs. with metastasis (2.14 vs. 2.05, *p* = 0.002 for *P16* CN; 19.1% vs. 4.7%, *p* = 0.004 for *P16* SCNamp; Table 1).

In addition, the average *P16* CN value and *P16* SCNamp-positive rate were significantly greater in poorly differentiated GC samples than in well- or moderately differentiated GC samples (1.96 vs. 1.76, *p* = 0.004 for *P16* CN; 22.9% vs. 45.1%, *p* = 0.006 for *P16* SCNdel; Table 1).

A significant difference in the prevalence of *P16* methylation was observed between pTNM I–II and III–IV stage GCs (0.0013 vs. 0.0036, *p* = 0.037; Table 1); a marginally significant difference also occurred in GCs with or without lymph node metastasis (0.0025 vs. 0.0013, *p* = 0.097; Table 1); and a difference in the *P16* methylation level in SMs was found between poorly and well/moderately differentiated patients (0.0021 vs. 0.0013, *p* = 0.018; Table 2).

**Figure 4:**
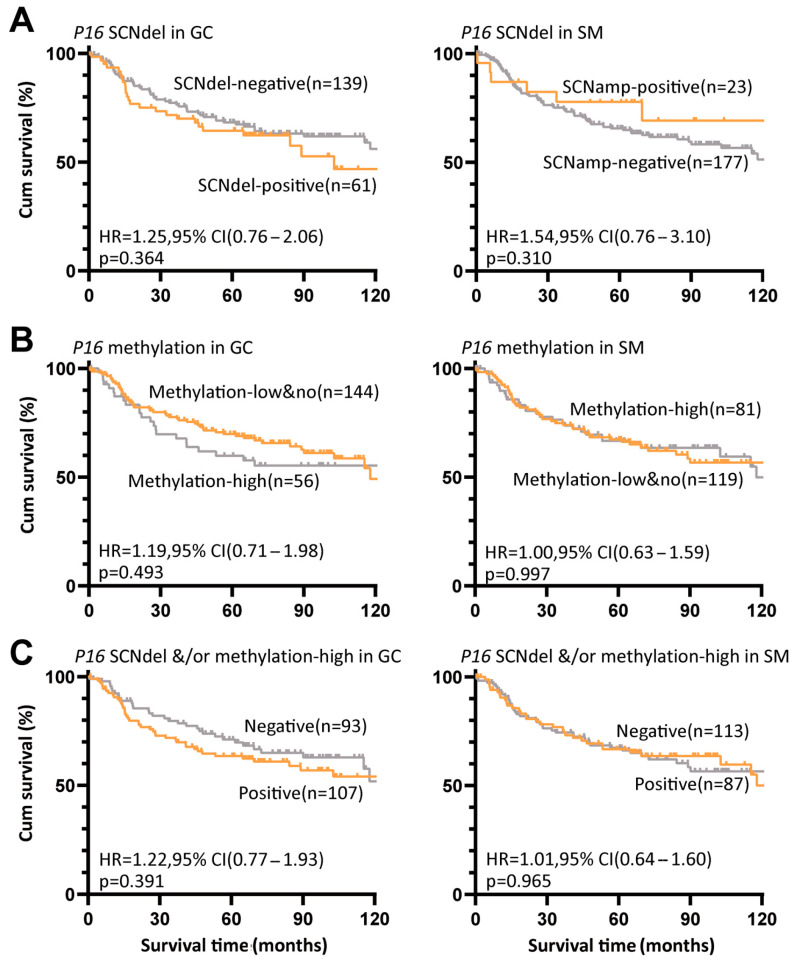
Overall survival curves for GC patients with different states of *P16* SCNVs and TSS-CGI methylation in gastric tissues according to K–M analyses. (**A**) Overall survival curves for patients with and without *P16* SCNdel in GCs or SCNamp in SMs; (**B**) Overall survival curves for patients with and without *P16* methylation-high in GCs or SMs; (**C**) Overall survival curves for patients with and without *P16* SCNdel &/or methylation-high in GCs or SMs. The hazard ratio (HR), 95% CI, and *p*-value are labeled according to log-rank univariate analysis.

In addition, we further investigated whether the overall survival of patients was associated with *P16* SCNVs and methylation. We found that the overall survival of patients with *P16* SCNamp-positive SMs or SCNdel-negative GCs was longer than that of patients with SCNamp-negative SMs or SCNdel-positive GCs, but the difference was not statistically significant (Figure 4A). Similar differences in overall survival were also observed between patients with *P16* methylation-high and P16 methylation-low/no GCs (Figure 4B). In the combination analysis, no synergistic effect was detected between *P16* SCNdel and methylation-high GCs or SMs (Figure 4C).

Taken together, our findings demonstrate that both *P16* SCNV and methylation in gastric samples are consistently associated with GC metastasis.

## 4. Discussions

Tumor suppressor genes are frequently inactivated both genetically and epigenetically in cancer genomes. Inactivation of one copy of tumor suppressor genes by germline point mutations is often subsequently accompanied by epigenetic inactivation of the wild-type copy of these mutant genes by DNA methylation in adult cells, which causes familial cancer [19]. For example, TSS-CGI hypermethylation serves as a frequent “second hit” for wild-type copies of these genes in inherited tumors and consequently causes hereditary diffuse GC and lobular breast cancer [20,21]. However, it is not known whether the SCNVs of tumor suppressor genes, including *P16*, are derived from TSS-CGI-methylated or nonmethylated genes in sporadic cancer genomes. Here, we report for the first time, to the best of our knowledge, that more *P16* SCNdel was detected in P16U GC samples, whereas more *P16* SCNamp was detected in P16M gastric samples. In addition, our findings indicate that GC metastasis is significantly associated with a decrease in *P16* CN and an increase in *P16* TSS-CGI methylation in gastric samples from GC patients.

As with the *CDH1* gene, one-allele inactivation of *CDKN2A*/*P16* leads to a high predisposition to familial cancers, indicating that it is a haplotype-insufficient gene [19,20,21]. *CDH1* is frequently inactivated in adult cells harboring a germline mutation of one *CDH1* allele [20,21]. Similar phenomena also occur in *P16*. For example, one *P16* allele is inactivated by a frame-shift mutation and another *P16* allele is inactivated by DNA methylation in human colon HCT116 cancer cells [22]. It is not known whether there is causality between frame-shift mutation and DNA methylation in *P16*.

Although we observed a correlation between *P16* SCNamp and TSS-CGI methylation in gastric tissues from 200 patients in this study, we do not know whether both *P16* SCNamp and TSS-CGI methylation occur at the same alleles or within the same cells. Whether these are two consequent or independent events is worthy of further study.

We previously reported that *P16* methylation or SCNdel increases the risk of GC metastasis [5,7,8]. In the present study, we simultaneously analyzed the states of *P16* methylation and SCNVs in GC and SM samples and reported that the levels of both *P16* CN and methylation correlated with GC lymph metastasis, suggesting a true role for *P16* inactivation in cancer metastasis. Our findings are consistent with studies using mouse models [7,11,23]. That *P16* SCNamp is coupled with DNA methylation might also contribute to the lack of significant difference in overall survival if amplified *P16* alleles are silenced by DNA methylation. A correlation between GC metastasis and *P16* CN or DNA methylation was observed at the same time point without interference from clinical management or socioeconomic status whereas a correlation between overall survival and *P16* alterations was observed at different time points with interference from these factors. The interference effect may account for the inconsistency between metastasis and overall survival.

Copy number deletion of *CDKN2A/B* is linked to early recurrence of meningioma. Although genome-wide DNA methylation and homozygous *CDKN2A/B* deletions are used for the classification of central nervous system tumors [24,25], whether a synergetic effect exists between *CDKN2A*/*P16* TSS-CGI methylation and heterozygous deletion and whether *P16* DNA methylation alone are prognosis factors are unknown.

In our previous study involving 80 GC patients [5], which was also included in the present study, we reported that the *P16* SCNamp in SMs correlated not only with a low risk of GC metastasis but also with long overall survival. However, in this study involving 200 GC patients, we observed that the *P16* SCNamp in SMs correlated only with a low risk of GC metastasis, but we did not observe a significant difference in overall survival. The small number of patients with *P16* SCNamp in SMs (23/200 = 11.5%) may account for the fluctuation.

## 5. Conclusions

Our study involving 200 GC patients revealed that *P16* SCNVs are coupled with the methylation status of the *P16* TSS-CGI in GC development and that SCNdel and TSS-CGI methylation complementarily inactivate *P16* and are associated with GC metastasis. While *P16* SCNdel coupled with TSS-CGI nonmethylation in gastric tissue samples, *P16* SCNamp coupled with TSS-CGI methylation in the samples implies that the amplified *P16* alleles may be silenced by DNA methylation during GC development.

## Figures and Tables

**Table 1:** Prevalence of somatic copy number variations (SCNVs) of the P16 gene in GC and SM samples from 200 patients.

			GC						SM					
			*P16* CN ^a^		Case Number for *P16* SCNV (%) ^b^				*P16* CN		Case Number for *P16* SCNV (%)			
		*n*	Mean ± SD	*p* Value ^c^	SCNdel	Diploid	SCNamp	*p* Value ^d^	Mean ± SD	*p* Value	SCNdel	Diploid	SCNamp	*p* Value
Age (yr)	≤60	80	1.95 ± 0.41	0.164	20 (25.0)	49 (61.3)	11 (13.7)	0.249	2.08 ± 0.23	0.508	6 (7.5)	65 (81.3)	9 (11.2)	0.895
	>60	120	1.86 ± 0.48		41 (34.2)	69 (57.5)	10 (8.3)		2.10 ± 0.22		7 (5.8)	99 (82.5)	14 (11.7)	
Sex	Male	140	1.89 ± 0.48	0.924	44 (31.4)	81 (57.9)	15 (10.7)	0.880	2.10 ± 0.22	0.833	8 (5.7)	115 (82.1)	17 (12.2)	0.736
	Female	60	1.90 ± 0.39		17 (28.3)	37 (61.7)	6 (10.0)		2.09 ± 0.22		5 (8.3)	49 (81.7)	6 (10.0)	
Neoadjuvant	Yes	64	1.95 ± 0.45	0.193	17 (26.6)	37 (57.8)	10 (15.6)	0.240	2.09 ± 0.27	0.857	4 (6.3)	49 (76.6)	11 (17.1)	0.224
chemotherapy	No	136	1.86 ± 0.46		44 (32.4)	81 (59.6)	11 (8.0)		2.09 ± 0.19		9 (6.6)	115 (84.6)	12 (8.8)	
GC location	Noncardiac	141	1.92 ± 0.43	0.192	41 (29.1)	84 (59.6)	16 (11.3)	0.715	2.09 ± 0.23	0.635	10 (7.1)	116 (82.3)	15 (10.6)	0.752
	Cardiac	59	1.83 ± 0.51		20 (33.9)	34 (57.6)	5 (8.5)		2.10 ± 0.21		3 (5.1)	48 (81.4)	8 (13.5)	
Differentiation ^e^	Well or mod.	71	1.76 ± 0.50	0.004	32 (45.1)	32 (45.1)	7 (9.8)	0.006	2.07 ± 0.27	0.456	7 (9.9)	54 (76.1)	10 (14.0)	0.186
	Poor	118	1.96 ± 0.43		27 (22.9)	77 (65.3)	14 (11.8)		2.10 ± 0.18		6 (5.1)	102 (86.4)	10 (8.5)	
pTNM stage	I–II	117	1.91 ± 0.45	0.558	34 (29.1)	71 (60.7)	12 (10.2)	0.842	2.12 ± 0.23	0.020	8 (6.8)	89 (76.1)	20 (17.1)	**0.012**
	III-IV	83	1.87 ± 0.46		27 (32.5)	47 (56.6)	9 (10.9)		2.05 ± 0.21		5 (6.0)	75 (90.4)	3 (3.6)	
Local invasion	T_1–2_	45	1.86 ± 0.46	0.573	17 (37.8)	24 (53.3)	4 (8.9)	0.480	2.10 ± 0.26	0.706	6 (13.3)	32 (71.1)	7 (15.6)	0.054
	T_3–4_	155	1.90 ± 0.46		44 (28.4)	94 (60.6)	17 (11.0)		2.09 ± 0.21		7 (4.5)	132 (85.2)	16 (10.3)	
Lymph	N_0_	94	1.91 ± 0.45	0.589	26 (27.7)	58 (61.7)	10(10.6)	0.708	2.14 ± 0.22	0.002	4 (4.3)	72 (76.6)	18 (19.1)	**0.004**
metastasis	N_1−X_^f^	106	1.88 ± 0.47		35 (33.0)	60 (56.6)	11 (10.4)		2.05 ± 0.21		9 (8.5)	92 (86.8)	5 (4.7)	
(Total)		200	1.89 ± 0.46		61 (30.5) ^g^	118 (59.0)	21 (10.5)		2.09 ± 0.22		13 (6.5)	164 (82.0)	23 (11.5)	

^a^ *P16* copy number (CN) in GC or SM was adjusted by that of WBC from the same patient; ^b^ somatic copy number variations in *P16* relative to WBC from the same patient; SCNdel or SCNamp: the difference in *P16* CN between WBC and tested tissue is ≤ 80% or ≥ 120% and *p* < 0.05 according to Student’s *t* test; ^c^ Student’s *t* test; ^d^ chi-square test; ^e^ no differentiation information for 11 cases; ^f^ including 7 cases with distant metastasis; ^g^ GC vs. SM, *p* < 0.001.

**Table 2:** The prevalence of *P16* methylation in gastric adenocarcinoma (GC) and surgical margin (SM) tissue samples from 200 patients.

			Prevalence of *P16* Methylation					
			GC			SM		
		*n*	Positive Rate (%)	Methylation Level (Median, 25–75%) ^a,b^	*p* Value ^c^	Positive Rate (%)	Methylation Level (Median, 25–75%)	*p* Value ^c^
Age (yr)	≤60	80	52 (65.0)	0.20 (0.08–0.71)	0.580	69 (86.3)	0.18 (0.07–0.37)	0.524
	>60	120	60 (50.0)	0.20 (0.07–2.19)		93 (77.5)	0.19 (0.07–0.39)	
Sex	Male	140	78 (55.7)	0.14 (0.07–1.48)	0.437	111 (79.3)	0.17 (0.07–0.33)	0.164
	Female	60	34 (56.7)	0.34 (0.08–0.98)		51 (85.0)	0.23 (0.07–0.62)	
Neoadjuvant	Yes	64	33 (51.6)	0.12 (0.06–0.52)	0.293	48 (75.0)	0.14 (0.05–0.27)	0.011
chemotherapy	No	136	79 (58.1)	0.32 (0.08–1.47)		114 (83.8)	0.21 (0.09–0.60)	
GC location	Noncardiac	141	85 (60.3)	0.20 (0.08–0.99)	0.724	119 (84.4)	0.19 (0.07–0.42)	0.383
	Cardiac	59	27 (45.8)	0.12 (0.07–3.79)		43 (72.9)	0.14 (0.06–0.31)	
Differentiation	Well or mod.	71	30 (42.3)	0.19 (0.08–1.65)	0.873	56 (78.9)	0.13 (0.06–0.29)	0.018
	Poor	118	75 (63.6)	0.20 (0.07–1.32)		99 (83.9)	0.21 (0.11–0.40)	
pTNM stage	I–II	117	64 (54.7)	0.13 (0.06–0.51)	0.037	90 (76.9)	0.21 (0.07–0.37)	0.950
	III–IV	83	48 (57.8)	0.36 (0.08–2.57)		72 (86.7)	0.17 (0.07–0.52)	
Local invasion	T_1–2_	45	25 (55.6)	0.13 (0.06–1.14)	0.663	39 (86.7)	0.21 (0.07–0.37)	0.842
	T_3–4_	155	87 (56.1)	0.20 (0.08–1.08)		123 (79.4)	0.18 (0.07–0.39)	
Lymph	N_0_	94	51 (54.3)	0.13 (0.07–0.52)	0.097	70 (74.5)	0.23 (0.07–0.37)	0.627
metastasis	N_1−X_	106	61 (57.5)	0.25 (0.07–2.56)		92 (86.8)	0.16 (0.07–0.52)	
(Total)		200	112 (56.0) ^d^	0.20 (0.07–1.06)		162 (81.0)	0.18 (0.07–0.37)	

^a^ for P16M samples; ^b^ ×10^−2^; ^c^ Mann–Whitney test; ^d^ GC vs. SM, *p* = 0.002.

## Data Availability

The original contributions presented in this study are included in the article. Further inquiries can be directed to the corresponding author.

## Supplementary Materials

The following supporting information can be downloaded at: https://www.mdpi.com/article/10.3390/cancers18101605/s1, Data File S1: Information for 200 patients.

## Abbreviations

The following abbreviations are used in this manuscript:

CN	Copy number
GC	gastric carcinoma
P16M	P16 methylation positive
P16U	P16 methylation negative
SCNamp	somatic copy number amplification
SCNdel	somatic copy number deletion
SCNVs	somatic copy number variation
SM	surgical margin
TSS-CGI	CpG islands around the transcription starting site
WBC	white blood cell

## References

[1] Sun Y., Deng D., You W.-C., Bai H., Zhang L., Zhou J., Shen L., Ma J.-L., Xie Y.-Q., Li J.-Y. (2004). Methylation of *p16* CpG Islands Associated with Malignant Transformation of Gastric Dysplasia in a Population-Based Study. Clin. Cancer Res..

[2] Cao J., Zhou J., Gao Y., Gu L., Meng H., Liu H., Deng D. (2009). Methylation of *p16* CpG Island Associated with Malignant Progression of Oral Epithelial Dysplasia: A Prospective Cohort Study. Clin. Cancer Res..

[3] Liu H., Liu X.-W., Dong G., Zhou J., Liu Y., Gao Y., Liu X.-Y., Gu L., Sun Z., Deng D. (2015). *P16* Methylation as an Early Predictor for Cancer Development from Oral Epithelial Dysplasia: A Double-blind Multicentre Prospective Study. EBioMedicine.

[4] Zhang X., Li P., Gan Y., Xiang S., Gu L., Zhou J., Zhou X., Wu P., Zhang B., Deng D. (2024). Driving effect of *P16* methylation on telomerase reverse transcriptase-mediated immortalization and transformation of normal human fibroblasts. Chin. Med. J..

[5] Luo D., Zhang B., Lv L., Xiang S., Liu Y., Ji J., Deng D. (2006). Methylation of CpG islands of *p16* associated with progression of primary gastric carcinomas. Lab. Investig..

[6] Fan Z., Zhou J., Tian Y., Qin Y., Liu Z., Gu L., Dawsey S.M., Wei W., Deng D. (2024). Somatic *CDKN2A* copy number variations are associated with the prognosis of esophageal squamous cell dysplasia. Chin. Med. J..

[7] Qiao J., Tian Y., Cheng X., Liu Z., Zhou J., Gu L., Zhang B., Zhang L., Ji J., Xing R. (2021). *CDKN2A* Deletion Leading to Hematogenous Metastasis of Human Gastric Carcinoma. Front. Oncol..

[8] Deng L., Zhou J., Sun Y., Hu Y., Qiao J., Liu Z., Gu L., Lin D., Zhang L., Deng D. (2024). *CDKN2A* somatic copy number amplification in normal tissues surrounding gastric carcinoma reduces cancer metastasis risk in droplet digital PCR analysis. Gastric Cancer.

[9] Merlo A., Herman J.G., Mao L., Lee D.J., Gabrielson E., Burger P.C., Baylin S.B., Sidransky D. (1995). 5′ CpG island methylation is associated with transcriptional silencing of the tumour suppressor *p16/CDKN2/MTS1* in human cancers. Nat. Med..

[10] Mao L., Lee J.S., Fan Y.H., Ro J.Y., Batsakis J.G., Lippman S., Hittelman W., Hong W.K. (1996). Frequent microsatellite alterations at chromosomes 9p21 and 3p14 in oral premalignant lesions and their value in cancer risk assessment. Nat. Med..

[11] Cui C., Gan Y., Gu L., Wilson J., Liu Z., Zhang B., Deng D. (2015). *P16*-specific DNA methylation by engineered zinc finger methyltransferase inactivates gene transcription and promotes cancer metastasis. Genome Biol..

[12] Serrano M., Hannon G.J., Beach D. (1993). A new regulatory motif in cell-cycle control causing specific inhibition of cyclin D/CDK4. Nature.

[13] Weng W., Zhang B., Deng D. (2024). P16^INK4A^ drives RB1 degradation by UTP14A-catalyzed K810 ubiquitination. iScience.

[14] Witkiewicz A.K., Venkata S.A.K., Knudsen E.S., Kumarasamy V. (2025). RB loss sensitizes triple-negative breast cancer to apoptosis induced by cellular stress. Cell Death Discov..

[15] Zamalloa L.G., Pruitt M.M., Hermance N.M., Gali H., Flynn R.L., Manning A.L. (2023). RB loss sensitizes cells to replication-associated DNA damage after PARP inhibition by trapping. Life Sci. Alliance.

[16] Gadhikar M.A., Zhang J., Shen L., Rao X., Wang J., Zhao M., Kalu N.N., Johnson F.M., Byers L.A., Heymach J. (2018). *CDKN2A/p16* Deletion in Head and Neck Cancer Cells Is Associated with CDK2 Activation, Replication Stress, and Vulnerability to CHK1 Inhibition. Cancer Res..

[17] Zhou J., Cao J., Lu Z., Liu H., Deng D. (2011). A 115-bp MethyLight assay for detection of *p16* (CDKN2A) methylation as a diagnostic biomarker in human tissues. BMC Med. Genet..

[18] Tian Y., Zhou J., Qiao J., Liu Z., Gu L., Zhang B., Lu Y., Xing R., Deng D. (2022). Detection of somatic copy number deletion of the CDKN2A gene by quantitative multiplex PCR for clinical practice. Front. Oncol..

[19] Esteller M., Fraga M.F., Guo M., Garcia-Foncillas J., Hedenfalk I., Godwin A.K., Trojan J., Vaurs-Barrière C., Bignon Y.J., Ramus S. (2001). DNA methylation patterns in hereditary human cancers mimic sporadic tumorigenesis. Hum. Mol. Genet..

[20] Machado J.C., Oliveira C., Carvalho R., Soares P., Berx G., Caldas C., Seruca R., Carneiro F., Sobrinho-Simöes M. (2001). E-cadherin gene (*CDH1*) promoter methylation as the second hit in sporadic diffuse gastric carcinoma. Oncogene.

[21] Corso G., Magnoni F., Molin M.D., Marino E., Nicosia L., Pesapane F., Noonan D.M., Albini A. (2025). Second-hit *CDH1* gene mechanisms in hereditary diffuse gastric and lobular breast cancer syndrome: Frequency and impact on tumorigenesis. Hum. Mol. Genet..

[22] Qin S., Li Q., Zhou J., Liu Z., Su N., Wilson J., Lu Z., Deng D. (2014). Homeostatic maintenance of allele-specific p16 methylation in cancer cells accompanied by dynamic focal methylation and hydroxymethylation. PLoS ONE.

[23] Chen S., Sanjana N.E., Zheng K., Shalem O., Lee K., Shi X., Scott D.A., Song J., Pan J.Q., Weissleder R. (2015). Genome-wide CRISPR Screen in a Mouse Model of Tumor Growth and Metastasis. Cell.

[24] Capper D., Jones D., Sill M., Hovestadt V., Schrimpf D., Sturm D., Koelsche C., Sahm F., Chaves L., Reuss D.E. (2018). DNA methylation-based classification of central nervous system tumors. Nature.

[25] Ippen F.M., Hielscher T., Patel A., Friedel D., Göbel K., Sievers P., Acker T., Snuderl M., Brandner S., Weller M. (2026). The prognostic impact of CDKN2A/B hemizygous deletions in meningioma. Neuro Oncol..

